# Hybrid Manufacturing of Acrylonitrile Butadiene Styrene (ABS) via the Combination of Material Extrusion Additive Manufacturing and Injection Molding

**DOI:** 10.3390/polym14235093

**Published:** 2022-11-23

**Authors:** Ke Gong, Handai Liu, Cheng Huang, Zhi Cao, Evert Fuenmayor, Ian Major

**Affiliations:** 1 PRISM Research Institute, Athlone Campus, Technological University of Shannon: Midlands and Midwest, N37 HD68 Athlone, Ireland; 2 School of Mechanical and Electronic Engineering, East China University of Technology, No. 418 Guanglan Road, Nanchang 330013, China

**Keywords:** additive manufacturing, materials extrusion, injection molding, hybrid manufacturing, tensile testing, infill density

## Abstract

Acrylonitrile Butadiene Styrene (ABS) is a common thermoplastic polymer that has been widely employed in the manufacturing industry due to its impact resistance, tensile strength, and rigidity. Additive manufacturing (AM) is a promising manufacturing technique being used to manufacture products with complex geometries, but it is a slow process producing mechanically inferior products when compared to traditional production processes like injection molding (IM). Thus, our hybrid manufacturing (HM) process combining materials extrusion AM and IM to create a single article was investigated in this study, in which eleven batches of specimens were made and extensively tested. These include the AM, IM, and hybrid manufactured (HYM) samples, in which the HYM samples were made by inserting AM substrates into the IM tool and were varied in infill density of AM preforms and geometries. The HYM samples outperformed AM parts in terms of mechanical performance while retaining customizability dependent on the HYM processing parameters, and the best mechanical performance for HYM samples was found to be comparable to that of IM samples, implying that the overmolding process in HM had primarily improved the mechanical performance of AM products. This work leads to a deeper knowledge of applications to confirm the optimal component fabrication in high design flexibility and mass production.

## 1. Introduction

Currently, increasing economic competition and the need to satisfy customers are compelling manufacturers to adhere to the mass customization (MC) principle, which aims to produce customized products at a price equivalent to that of mass-produced goods [1]. MC consists of two basic components: product variety, which is defined as the variety of goods produced and available on the market due to the manufacturing chain [2], and process variety, which refers to the processing variants used to validate the variant fabrications [3]. In this scenario, the manufacturing technology with the capacity to make customized goods earns public favor. Thus, 3D Printing (3DP), also known as additive manufacturing (AM), has been extensively improved and applied in a variety of fields, including chemical education [4], strain sensors [5], magnetic soft material fabrications [6], household appliances [7], the aerospace industry [8], and studies in the field of materials [9], while keeping outstanding manufacturing flexibility [10,11]. In addition, 3DP is able to directly build 3D structures in different sizes with a specific design framework, which conventional manufacturing techniques cannot achieve, like injection molding (IM) [12,13,14,15,16,17]. Fused deposition modeling (FDM) is the most widely utilized technique that begins with a 3D model created using Computer Aided Design (CAD) software, which is then converted to a stereolithography (STL) extension file. Each model is then cut into slices containing the specifics of its corresponding levels [18]. In order to create the objects, thermoplastic materials in the form of filaments are first melted in the hot end and then extruded onto the building plate [19,20,21,22,23,24,25]. Furthermore, a variety of regularly used materials, such as Acrylonitrile Butadiene Styrene (ABS) [17] and Polylactic Acid (PLA) [26], can be considered in FDM based on the needed properties. In addition to diverse material options, the ease of operation, low cost, and environmental friendliness of FDM have contributed to its widespread use in a variety of industries [7,8,9,27,28,29]. Several researchers, however, have emphasized a common disadvantage of FDM-fabricated objects, namely that their performance is inferior to that of IM-fabricated objects [27,30,31].

IM is a manufacturing process that creates plastic parts by forcing molten polymer under high pressure into a mold cavity with the desired final physical form of the item [32]. A non-return mechanism at the front of the screw prevents the melt from moving backward. The molten material is carried through the reciprocating screw to a region before the nozzle generated by the displacement of the screw away from the nozzle by the material collecting in front of the screw. The movement of the screw injects molten material into the mold to fill the cavity. Initially, the pieces will be allowed to cool within the molding tool for a period of time dictated by the material’s nature or the required final qualities. This is followed by the mold opening through which the part is expelled [33]. The mold shuts, and the procedure restarts [34]. IM can make numerous plastic products, including bottle caps, some important daily tools (one-piece chairs and tiny tables), storage containers, and mechanical parts (gears). These factors make it one of the most widely used manufacturing processes, which can be attributed to two aspects: low running costs in the process of mass fabrication of parts (a durable mold that can be used for extended periods of time) and producing a large number of identical specimens in a short amount of time with excellent dimensional accuracy. However, there are still several obstacles to using IM, such as the lengthy installation period and the difficulty of making design modifications cheaply. Researchers have offered several methods for producing hybrid samples with the importation of IM’s benefits but without the downsides [30,35,36].

In this regard, a type of hybrid manufacturing (HM) combining FDM and IM in a single location has been investigated. This hybrid strategy is notable for its exceptional FDM and IM capabilities due to the high design flexibility of the FDM technique, which is a significant advantage over traditional IM, and the boosted manufacturing efficiency, which is a big challenge for the FDM technique and has synergistic effects on highly complex geometric components. Dr. Fuenmayor et al. [36] used this HM technology in a pharmaceutical setting, showing that this method reduces production time and improves customization. Mr. Rajamani examined the bonding between the FDM preforms and IM components and investigated several ideas to improve the bonding [37]. In the current investigation, overmolding of tensile specimens happened over the whole length of the FDM substrates for all samples produced by HM. These preforms were varied in infill density and geometry and then inserted into the mold cavity to fabricate all HYM specimens. To establish the mechanical performance of HYM samples, tensile and morphological tests were conducted to determine the effect of HM in the finished specimens. The findings presented in this paper serve as a starting point for determining and exploiting the effects of strategies accessible via HM on samples based on the varied parameter sets employed in the study to increase the number of tools available for simultaneously enhancing personalization and mechanical properties.

## 2. Materials and Methods

### 2.1. Materials

ABS filament with a diameter of 1.75 mm used in the FDM stage of this project was supplied by Real Filament Company (Wateringen, The Netherlands). The virgin ABS pellets utilized in the IM processing were supplied by LOTTE Advanced Material Corporation (Seoul, Korea). The ABS pellets were first dried at 70 °C for 4 h prior to the IM processing.

### 2.2. Sample Preparation

**Figure 1:**
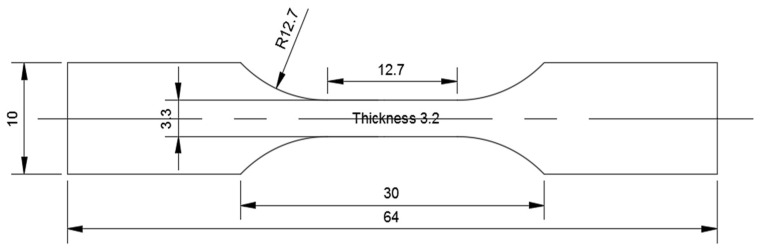
Detailed dimensions of tensile bar specimens produced for characterization in this study (unit in millimeters).

Three sets of samples (FDM, IM, and HYM) were produced in this study to compare their mechanical performances. All manufactured specimens in this study were fabricated following the ASTM-D638-3 Standard, and the dimensions of the fabricated samples are shown in Figure 1.

Three batches of FDM samples and seven kinds of inserts applied in the overmolding processing were produced by the FDM technique using an Orion Delta 3D Printer (SeeMe CNC, Ligonier, IN, USA). The software suite used to control the printer was Simplify 3D Software Version-4.1.1 (Cincinnati, OH, USA). All CAD models were designed using SolidWorks 2016 Software (Dassault Systèmes, Waltham, MA, USA). The main parameters applied in the FDM processing were selected based on previously published literature [17,38,39,40,41] and shown in Table 1. The model sizes of all FDM inserts were set as 105% size due to the material shrinkage in the sample fabrications. The parameters employed in the FDM technique were tested via producing high-quality manufacturing resolution test plates (pins and voids) prior to the fabrication of FDM specimens and inserts.

**Figure 2:**
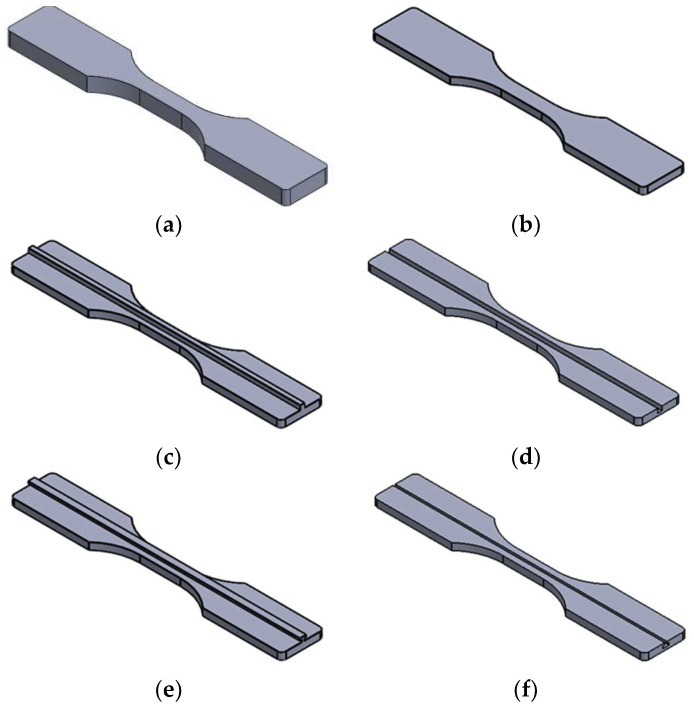
CAD models of all FDM articles.

The printing thickness, also known as the layer height of the prototyping process, was set at 0.1 mm in this study, and the FDM parts/inserts are shown in Figure 2: (a) normal FDM sample; (b) half-thickness without joint; (c) half-thickness male cube type joint; (d) half-thickness female cube type joint; (e) half thickness male T type joint; and (f) half thickness female T type joint.

The IM processing was carried out via a Babyplast 6/P injection molding machine (Rambaldi Company, Molteno, Italy), whose working parameters are listed in Table 2 below. The required shot size for IM processing was set and determined by the volume of material necessary per shot to fill all sections, including runners, gates, and cavities [42]. All IM settings were optimized prior to the production of samples.

**Figure 3:**
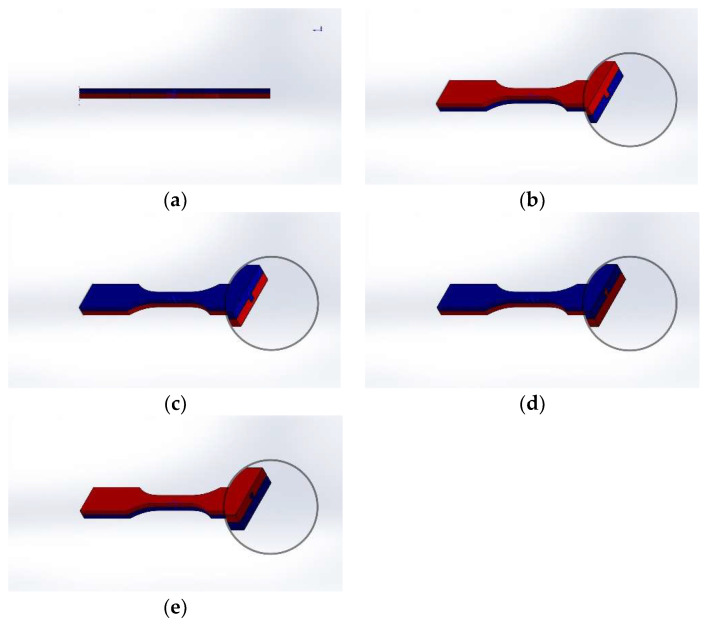
CAD design for finished HYM samples: (**a**) NJ series; (**b**) MC 50; (**c**) FC 50; (**d**) MT 50; and (**e**) FT 50. Red represents the FDM preform, and blue the IM substrate.

Eleven batches of samples were prepared in this study to compare the difference between the FDM, IM, and HYM samples. Three different infill densities (25%, 50%, and 75%) were used to determine the impact of the infill density on the performance of the samples. In addition, four joint configurations, along with the batches without joint configuration applied in the HYM samples, were selected to evaluate the effect of interfacial surfaces on the final samples. Each sample set consisted of twelve specimens for the defined group of process parameters, with a total of 132 samples in the study. The finished HYM specimens are shown in Figure 3.

### 2.3. Tensile Test for the Samples

All tensile tests were carried out using a Lloyd LRX Material Testing Machine (JLW Instruments Corporation, Chicago, IL, USA) at a movement speed of 5 cm/min at room temperature, where the tensile stress and Young’s Modulus of all parts were collected and analyzed using the NEXYGEN™ software to obtain their individual average values and standard deviations [43].

### 2.4. Dimensional Accuracy Study

A Nikon Digital Microscope (Firmware ShuttlePix P-MFSC) from Japan with a magnification of (*20) was used to measure the parameters of fabricated samples and observe their macrostructures, providing the size accuracy of parts produced in this study.

### 2.5. Statistical Analysis

Minitab 18 software (Minitab LLC, State College, Pennsylvania, USA) was utilized to analyze the tensile stress and Young’s Modulus through analysis of variance (ANOVA) [44] and to figure out the impact of joint configuration on HYM samples’ mechanical performances [9,45]. The *p*-value was defined at 0.05 [7].

### 2.6. Composite Morphology

A Mira FE scanning electron microscopy (SEM) was used to illustrate the microstructure and dispersion morphology of the samples after testing on the fracture areas, with a 20 kV acceleration voltage and the magnification of (*50) to visualize the dispersion via a backscattered electron detector. Prior to the SEM investigations, the samples observed were gold coated using a Baltec SCD 005 Sputter Coater [45].

## 3. Results and Discussion

In this study, the most important batches are those manufactured according to the MC principle, taking into account the sustainability of the process and the mechanical integrity of the parts. The AM-IM hybrid manufacturing process achieves the MC strategy in specimen fabrication: the FDM machine generates multiple inserts with specific properties (infill density and shape), and the IM process completes the creation of HYM specimens. FDM was used to fabricate parts that would be put into the IM tool with a high degree of geometrical freedom. Additionally, IM completes the HYM specimens at faster production rates than FDM alone.

Fabrication time of specimens was the first concern in this investigation, with a single tensile specimen fabricated using FDM taking an average of 12 min to build, depending on processing parameters, particularly infill density, whereas the IM process fabricates two specimens in 1.5 min (this is the molding cycle length). In this investigation, each batch comprised 12 specimens, and it took around 144 min to build each batch of FDM specimens but just 9 min to fabricate each batch of IM specimens. In contrast, the current HYM method took 72 min for insert fabrication plus 9 min for IM, for a total of 81 min per batch via HM, indicating that HM manufacturing requires less time than FDM. In this investigation, the customizability of 3D printing allowed for the manufacture of different inserts during the overmolding step resulting in the inclusion of multiple batches of HYM samples, which are listed in Table 3.

### 3.1. Dimensional Accuracy

**Figure 4:**
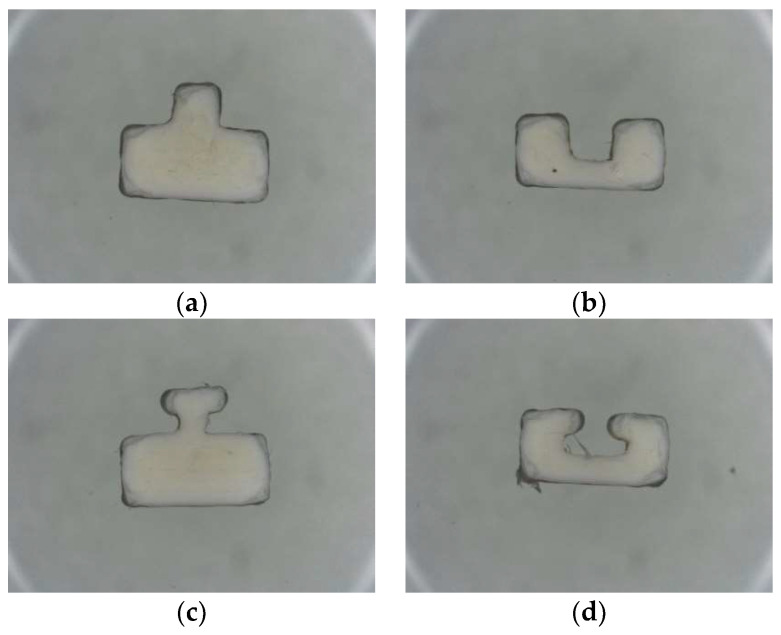
Cross sections of FDM inserts: (**a**) MC 50, (**b**) FC 50, (**c**) MT 50, and (**d**) FT 50.

All fabricated specimens for the tensile test were observed via a Nikon ShuttlePix P-MFSC machine in which gauge length, specimen thickness, and specimen length were considered and measured following the ASTM-638-3 standard: the dimensions are shown in Table 4. The cross sections of joint configurations for FDM inserts, as well as the parameters of the gauges for the final samples, are shown below in Figure 3 and Figure 4, respectively.

### 3.2. Ultimate Tensile Strength and Stiffness

The test results, including mean and standard deviations, of the maximum tensile stress/strengths (σ) and Young’s Modulus (E) for all ABS samples are tabulated in Table 5 and Table 6: Table 5 compares full FDM and IM samples, and the HYM samples are presented in Table 6.

Table 5 displays the tensile test results for specimens fabricated through FDM or IM only, where all average maximum tensile stress and Young’s Modulus progressively fell as the infill density decreased in the FDM specimens. The best mechanical property was observed in the IM batch, which is in line with previous publications [46,47,48] that samples with higher infill density will show greater mechanical performance than lower-infill density FDM parts. In addition, a high value of maximum tensile stress for the IM batch can be found here, which can be explained by the high-density internal structure and high material compaction of the IM specimen, offering a stable withstanding while loaded with the tensile.

Table 6 compares the mechanical properties of all HYM samples during the tensile test, in which FT 50 showed the highest value of average tensile strength, 48 MPa, almost equal to that for the IM batch in Table 5, while the average tensile strength of FC 50 was slightly lower than 48 MPa, with a value of 47.42 MPa. MT 50 showed the worst average tensile strength of all HYM samples with a value of 42.62 MPa but was remarkably better than all FDM samples. In relation to the value of Young’s Modulus, MT 50 carried the highest amongst HYM samples (854.54 MPa), while the worst belonged to NJ 75 (690.35 MPa).

### 3.3. Stress-Strain Behaviors

**Figure 5:**
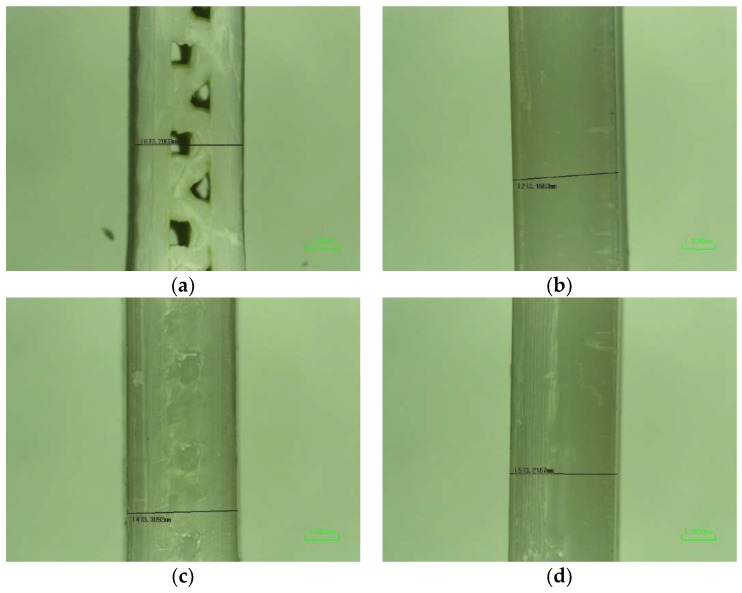

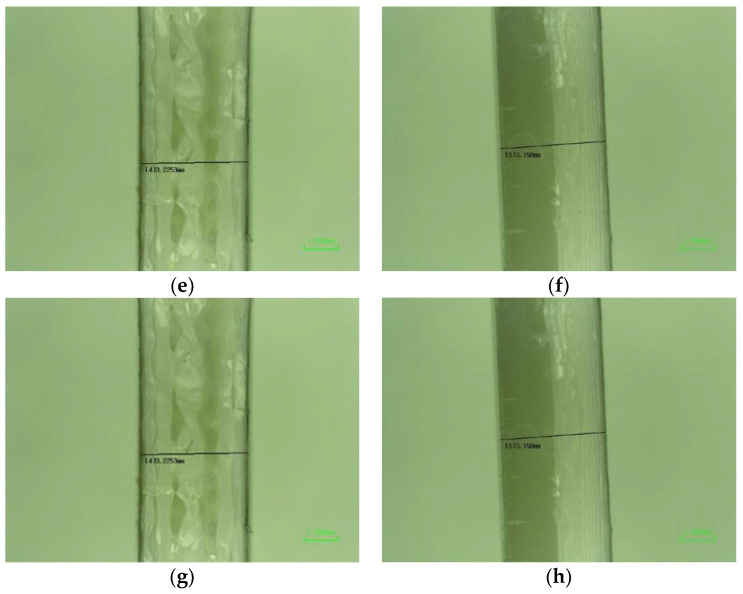
Images by Nikon Microscope for macrostructures and parameters of samples: (**a**) FDM 50, gauge width for 3.2083 mm; (**b**) FDM 50, thickness for 3.1438 mm; (**c**) IM, gauge width for 3.4501 mm; (**d**) IM, thickness for 3.1663 mm; (**e**) NJ 50, gauge width for 3.3092 mm; (**f**) NJ 50, thickness for 3.2167 mm; (**g**) FC 50, gauge width for 3.2253 mm; and (**h**) FC 50, thickness for 3.158 mm.

**Figure 6:**
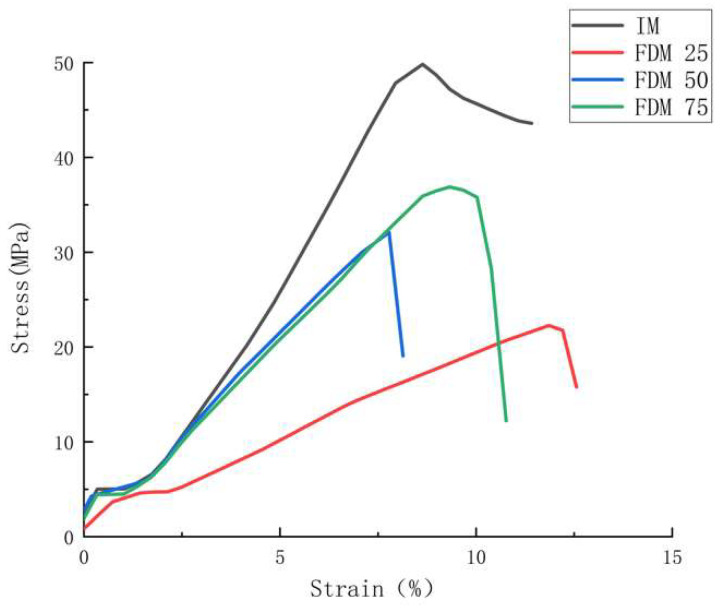
Representative stress-strain curves for tensile test amongst FDM and IM batches.

**Figure 7:**
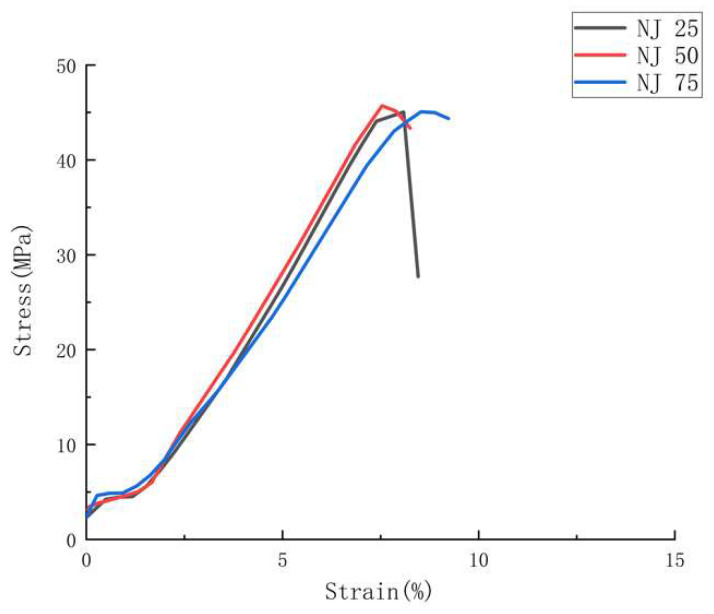
Representative stress-strain curves for tensile test amongst NJ batches.

The representative stress-strain curves, Figure 5, Figure 6 and Figure 7, show the stress-strain behaviors for all ABS specimens under tensile loading.

Figure 6 shows representative stress-strain curves for all FDM and IM batches in which the maximum tensile strength varies from 22.85 to 49.95 MPa, which clearly depicts an increasing tendency for tensile strength with the rise of infill density, agreeing with the result in Table 5. In addition, FDM 25 shows the highest value of strain amongst all FDM samples, reaching around 12%. These phenomena can be explained by two statements: (1) parts with higher infill density will be more rigid and have fewer cavities [48], which makes FDM 75 show the greatest mechanical performance amongst the FDM samples; (2) the higher degree of internal defects for the lower infill-density samples will result in greater elongation at the break due to the poor bonding force amongst fiber in FDM samples [48].

Moreover, IM’s outstanding mechanical performance can be found in the IM batch based on Figure 5 and Table 5, with a high-value difference when compared to batches 1–3. This can be explained by two factors: (1) the high-density structure of ABS samples, in addition to the strong polymer chain, can be created through IM processing, which agrees with some previous studies [8,31,46]; (2) the layer-by-layer formation in FDM parts results in the stress concentration at the fusion line, which further aligns with previous findings [49,50,51].

As for the tensile results related to the NJ 25, NJ 50, and NJ 75 batches, what can be found firstly in Figure 7 is that these samples show their significantly higher tensile stress if compared with all batches of FDM samples in Figure 5. Even the batch with the greatest tensile performance amongst FDM samples (FDM 75) still shows worse tensile stress than the worst batch amongst the NJ group (37.91 MPa < 45.09 MPa), showing HYM samples have superior tensile properties compared to FDM samples. In addition, Figure 7 shows a different condition in relation to the infill density of NJ, which is fabricated from 50% infill density inserts, and shows the greatest tensile performance compared with NJ 25 (25% infill) and NJ 75 (75% infill). This can be attributed to the outstanding ductility of ABS material, offering the strongest fusion bonding upon a 50% infill density insert.

**Figure 8:**
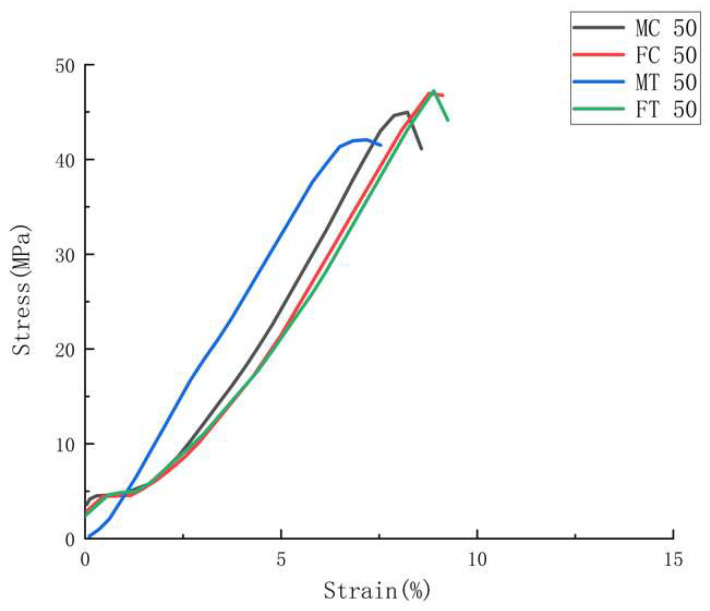
Representative stress-strain curves for tensile test amongst all HYM samples with joint configurations.

**Figure 9:**
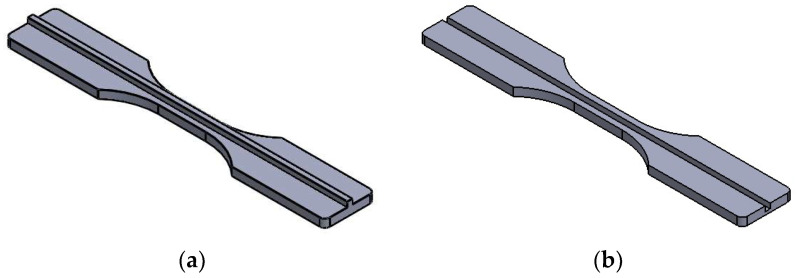
Schematic graph of cube inserts applied in overmolding processing: (**a**) male cube; (**b**) female cube.

Figure 8 shows representative stress-strain curves for the HYM samples with joint configurations, through which the impact of joint configurations on the HYM samples’ mechanical performances can be compared. The joint configurations’ effort in mechanical performance can be mainly categorized into two aspects, the type (male and female) and geometry (cube and T shapes). It can be found in Figure 8 and Table 6 that the female joint configuration resulted in greater tensile stress than the male joint configuration (47.42 MPa > 45.29 MPa for cubes and 48 MPa > 42.62 MPa for T joints). In relation to the brittle and ductile performance during fracture, FC 50 and FT 50 had more ductile performances (8–10% strain), while MC 50 and FC 50 displayed more brittle behaviors (6–8% strain). In addition, an interesting phenomenon can be found that the standard deviations for the female joints on the maximum stress value and Young’s Modulus were lower than those for the male joints, respectively, which indicates a greater reliability on female joints than the male joint [52]. Simultaneously, the result of ANOVA has been shown in Table 7, where the degree of freedom, sum of squares, mean square, f-ratio, and *p*-value are included. The *p*-values of the ultimate tensile stress and Young’s Modulus are less than 0.05, indicating significant impacts of joint configuration on tensile stress and Young’s Modulus. The phenomena can be attributed to two main factors and have been discussed as follows: 1. a female joint configuration (cube), shown in Figure 9b, would offer a stronger mechanical interlock based on the overmolding processing than the male cube shown in Figure 9a; 2. a higher volume of the molten polymer was able to enter the female joints compared to the male ones. This illustrates that a greater emphasis on tailored characteristics is required for an increase in the tensile strength of HYM specimens and may be used in certain elements, such as assembly-based toys or parts such as LEGO brand, gears in the manufacturing industry, and the cup holder tray in a coffee machine.

### 3.4. Fractographic Analysis of the Tensile Failure of ABS Samples

**Figure 10:**
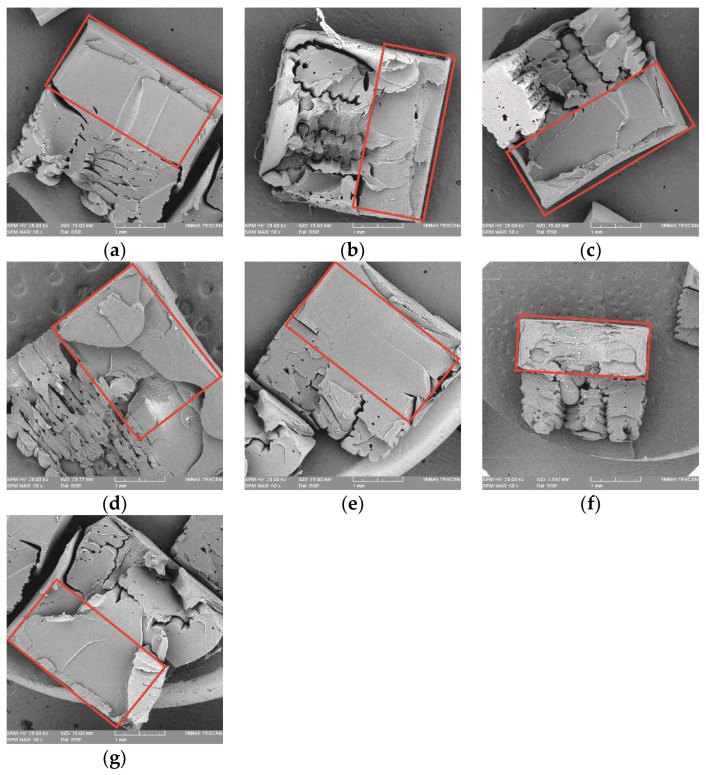
SEM images showing the fracture sections of specimens, in which the IM part is marked with a red square: (**a**) NJ25; (**b**) NJ50; (**c**) NJ75; (**d**) MC50; (**e**) FC50; (**f**) MT50; and (**g**) FT50.

Figure 10 shows SEM images of the fractured tensile interfaces of all HYM specimens. The comparison of fractured morphologies displays that both the infill density of FDM inserts and the joint configurations employed in the HM technique affect the tensile performance. Figure 10a–c shows that several cracks could be found during the tensile loading, but NJ 50 showed more than NJ 25 and NJ 75, especially in the fractured interlayer area, which proved the greatest mechanical performance of the HYM samples with 50% infill density inserts. During the tensile test, the first few cracks were generated in the overmolded polymer, which possessed a greater hardness than the FDM area to inhibit the fracture. However, more cracks were created to keep the tensile loading process. More tensile loading would be required and result in more new cracks, and finally, the tensile stress increased [52]. Further, the damage in the MC 50 and FC 50 showed more brittle morphologies due to the cube-shape joint configuration than the MT 50 and FT 50, which agrees with the results obtained in the tensile test. Failures for these HYM samples were in relation to the trans-interlayer tensile failure by the fracture of polymer fusion bonds themselves, leading to ductile morphologies and greater stiffness. The phenomena of Young’s Modulus and strain deliver a solution in certain bespoken appliances requiring higher stiffness in several fields, such as frames of bicycles, chairs, television supports, and specific assembled parts in aircraft.

## 4. Conclusions

Through comparative tensile tests on FDM, IM, and HYM samples, the effect of AM-IM hybrid manufacturing on the mechanical properties of ABS samples has been investigated in this work. Consideration was given to the infill density of inserts (25 percent, 50 percent, and 75 percent) and joint configurations, both of which were employed in HM processing (four types of joint configurations along with one series with no joint configurations).

The study provides evidence for the following claims:

(1) Mechanical properties of IM samples surpassed those of other samples due to the nature of the IM process, which results in strong material compaction and enhanced internal morphology.

(2) The infill density of FDM inserts had a significant impact in determining the mechanical performance of HYM samples, with the good flexibility of ABS material providing superior mechanical performance for HYM samples with a medium infill density (50 percent for HYM samples).

(3) The female T-shape joint showed the strongest interlayer bonding and the biggest interlayer surface compared to the other joint types, resulting in the greatest tensile performance compared to other HYM samples and FDM specimens. This makes HM particularly attractive for employment in manufacturing products with bespoken features and great mechanical characteristics, such as some butterfly models fabricated on the fridge handler, hence addressing one of the drawbacks of FDM specimens’ low mechanical properties if compared to IM parts.

(4) Manufacturing cost is a big topic in the market; HYM samples demonstrated their ability to achieve cost reduction as well as execute a facile, personalized feature, which is present in all HYM samples. A reduction in manufacturing time can be found in specimen fabrications using the HM technique compared to the FDM technique. This expands the availability in manufacturing of goods with customized characteristics and high quality at a reasonable cost compared to the high expense of mold design and manufacture in the IM technique.

However, the absence of research into the injection molding pressure used in the overmolding process, the printing speed, build material, and temperature used in insert fabrications necessitates future research to expand our understanding and exploitation of this manufacturing technology.

## Figures and Tables

**Table 1:** FDM parameters applied in the study.

FDM Parameters	
Printing speed (mm/min)	3000
Infill orientation (degrees)	±45
Outline overlap (%)	20
Number of top solid layers	1
Number of bottom solid layers	1
Number of outline/perimeter shells	2
Infill extrusion width (%)	100
Minimum infill length (mm)	5
Extruder temperature (°C)	210
Printing bed temperature (°C)	110
Retraction distance (mm)	8.4
Retraction vertical lift (mm)	0.1
Retraction speed (mm/min)	3600
Diameter of nozzle (mm)	0.5

**Table 2:** Parameters of IM applied in the study.

Injection Times and Settings	
Shot size (mm)	40 for IM batch, 30 for HYM batches
Cooling time (sec)	20
Plasticizing zone temperature (°C)	190
Chamber temperature (°C)	180
Nozzle temperature (°C)	170
Mold temperature (°C)	50
1st injection pressure (bar)	100
1st injection pressure time (sec)	3.5
2nd injection pressure (bar)	50
2nd injection pressure time (sec)	3
2nd pressure setting (mm)	3
Decompression (mm)	2
Injection speed (%)	95
2nd injection speed (%)	50
2nd Speed point (mm)	4

**Table 3:** Range of samples.

Batch Name	Type of Samples/Inserts	Infill Density
FDM 25	FDM Sample	25%
FDM 50	FDM Sample	50%
FDM 75	FDM Sample	75%
IM	IM Sample	100%
NJ 25	Half of Thickness (No Joint Configuration)	25%
NJ 50	Half of Thickness (No Joint Configuration)	50%
NJ 75	Half of Thickness (No Joint Configuration)	75%
MC 50	Half of Thickness Male Cube Joint	50%
FC 50	Half of Thickness Female Cube Joint	50%
MT 50	Half of Thickness Male T Joint	50%
FT 50	Half of Thickness Female T Joint	50%

**Table 4:** Parameters of sections for all samples.

Parameter	IM Batch	FDM Batches	HYM Batches
Thickness (mm)	3.2 ± 0.1	3.2 ± 0.1	3.2 ± 0.1
Total Length (mm)	64 ± 0.2	64 ± 0.2	64 ± 0.2
Gauge Width (mm)	3.3 ± 0.2	3.3 ± 0.1	3.3 ± 0.1
Width at two ends (mm)	10 ± 0.2	10 ± 0.2	10 ± 0.2

**Table 5:** Average tensile test results for all FDM and IM batches, whose standard deviations have been shown in the brackets.

Batch Name	σ (MPa)	E (MPa)
FDM 25	22.85 ± 2.97	446.42 ± 86.86
FDM 50	32.26 ± 1.56	675.97 ± 79.17
FDM 75	37.91 ± 4.66	689.19 ± 95.01
IM	49.95 ± 0.61	887.49 ± 136.19

**Table 6:** Average tensile test results for all HYM samples, whose standard deviations have been shown in the brackets.

Batch Name	σ (MPa)	E (MPa)
NJ 25	45.09 ± 2.90	724.96 ± 47.37
NJ 50	46.62 ± 0.87	745.03 ± 24.63
NJ 75	45.62 ± 1.23	690.35 ± 68.90
MC 50	46.30 ± 0.81	746.07 ± 60.62
FC 50	47.42 ± 0.97	737.62 ± 15.43
MT 50	42.62 ± 2.14	861.23 ± 64.63
FT 50	48.00 ± 1.07	727.92 ± 37.09

**Table 7:** ANOVA of the model for Ultimate Tensile Stress and Young’s Modulus. Degree of Freedom for DF, Sum of Squares for SS, Mean Square for MS, F-Ratio for F, and *p*-Value for *p*.

		Ultimate Tensile Stress				Young’s Modulus			
Source	DF	SS	MS	F	*p*	SS	MS	F	*p*
Model	4	176.91	44.23	24.66	0	121,323	30,331	20.01	0
Residual	45	80.7	1.793			68,221	1516		
Total	49	257.61				189,544			

## Data Availability

The data presented in this study are contained within the article.

## References

[1] Daaboul J., da Cunha C., Bernard A., Laroche F. (2011). Design for mass customization: Product variety vs. process variety. CIRP Ann..

[2] Ulrich K.T. (1995). The role of product architecture in the manufacturing firm. Res. Policy.

[3] Du X. (2000). Architecture of Product Family for Mass Customization.

[4] Alderighi T., Giorgi D., Malomo L., Cignoni P., Zoppè M. (2021). Computational design, fabrication and evaluation of rubber protein models. Comput. Graph..

[5] Huang P., Xia Z., Cui S. (2018). 3D printing of carbon fiber-filled conductive silicon rubber. Mater. Des..

[6] Ganguly S., Margel S. (2022). 3D printed magnetic polymer composite hydrogels for hyperthermia and magnetic field driven structural manipulation. Prog. Polym. Sci..

[7] Byberg K.I., Gebisa A.W., Lemu H.G. (2018). Mechanical properties of ULTEM 9085 material processed by fused deposition modeling. Polym. Test..

[8] Weng Z., Wang J., Senthil T., Wu L. (2016). Mechanical and thermal properties of ABS/montmorillonite nanocomposites for fused deposition modeling 3D printing. Mater. Des..

[9] Hutmacher D.W., Schantz T., Zein I., Ng K.W., Teoh S.H., Tan K.C. (2001). Mechanical properties and cell cultural response of polycaprolactone scaffolds designed and fabricated via fused deposition modeling. J. Biomed. Mater. Res..

[10] Mozaffar M., Ndip-Agbor E., Lin S., Wagner G.J., Ehmann K., Cao J. (2019). Acceleration strategies for explicit finite element analysis of metal powder-based additive manufacturing processes using graphical processing units. Comput. Mech..

[11] Yang Y., Gong Y., Li C., Wen X., Sun J. (2020). Mechanical performance of 316 L stainless steel by hybrid directed energy deposition and thermal milling process. J. Mater. Process. Technol..

[12] Casavola C., Cazzato A., Moramarco V., Pappalettere C. (2016). Orthotropic mechanical properties of fused deposition modelling parts described by classical laminate theory. Mater. Des..

[13] Domingo-Espin M., Puigoriol-Forcada J.M., Granada A.A.G., Llumà J., Borros S., Reyes G. (2015). Mechanical property characterization and simulation of fused deposition modeling Polycarbonate parts. Mater. Des..

[14] Melenka G.W., Cheung B.K.O., Schofield J.S., Dawson M.R., Carey J.P. (2016). Evaluation and prediction of the tensile properties of continuous fiber-reinforced 3D printed structures. Compos. Struct..

[15] Stansbury J.W., Idacavage M.J. (2016). 3D Printing with Polymers: Challenges among Expanding Options and Opportunities. Dent. Mater..

[16] Sugavaneswaran M., Arumaikkannu G. (2015). Analytical and experimental investigation on elastic modulus of reinforced additive manufactured structure. Mater. Des..

[17] Tymrak B.M., Kreiger M., Pearce J.M. (2014). Mechanical properties of components fabricated with open-source 3-D printers under realistic environmental conditions. Mater. Des..

[18] Wicaksono S.T., Ardhyananta H., Pambudi A.I. (2017). Influence of internal geometri on mechanical properties of 3D printed polylactic acid (PLA) material. Int. J. Eng. Sci. Res. Technol..

[19] Alafaghani A.A., Qattawi A. (2018). Investigating the effect of fused deposition modeling processing parameters using Taguchi design of experiment method. J. Manuf. Process..

[20] Christiyan K.G.J., Chandrasekhar U., Venkateswarlu K. (2016). A study on the influence of process parameters on the Mechanical Properties of 3D printed ABS composite. IOP Conf. Ser. Mater. Sci. Eng..

[21] Sanatgar R.H., Campagne C., Nierstrasz V. (2017). Investigation of the adhesion properties of direct 3D printing of polymers and nanocomposites on textiles: Effect of FDM printing process parameters. Appl. Surf. Sci..

[22] Huang B., Singamneni S. (2014). Raster angle mechanics in fused deposition modelling. J. Compos. Mater..

[23] Ning F., Cong W., Hu Y., Wang H. (2016). Additive manufacturing of carbon fiber-reinforced plastic composites using fused deposition modeling: Effects of process parameters on tensile properties. J. Compos. Mater..

[24] Ning F., Cong W., Qiu J., Wei J., Wang S. (2015). Additive manufacturing of carbon fiber reinforced thermoplastic composites using fused deposition modeling. Compos. Part B Eng..

[25] Wei X., Li D., Jiang W., Gu Z., Wang X., Zhang Z., Sun Z. (2015). 3D Printable Graphene Composite. Sci. Rep..

[26] Drummer D., Cifuentes-Cuéllar S., Rietzel D. (2012). Suitability of PLA/TCP for fused deposition modeling. Rapid Prototyp. J..

[27] Dawoud M., Taha I., Ebeid S.J. (2016). Mechanical behaviour of ABS: An experimental study using FDM and injection moulding techniques. J. Manuf. Process..

[28] Kalita S.J., Bose S., Hosick H.L., Bandyopadhyay A. (2003). Development of controlled porosity polymer-ceramic composite scaffolds via fused deposition modeling. Mater. Sci. Eng. C.

[29] Zhang Y., Chou K. (2008). A parametric study of part distortions in fused deposition modelling using three-dimensional finite element analysis. Proc. Inst. Mech. Eng. Part B J. Eng. Manuf..

[30] Boros R., Rajamani P.K., Kovacs J.G. (2019). Combination of 3D printing and injection molding: Overmolding and overprinting. Express Polym. Lett..

[31] Lay M., Thajudin N.L.N., Hamid Z.A.A., Rusli A., Abdullah M.K., Shuib R.K. (2019). Comparison of physical and mechanical properties of PLA, ABS and nylon 6 fabricated using fused deposition modeling and injection molding. Compos. Part B Eng..

[32] Sreedharan J., Jeevanantham A. (2018). Analysis of Shrinkages in ABS Injection Molding Parts for Automobile Applications. Mater. Today Proc..

[33] Todd R.H., Allen D.K. (1994). Manufacturing Processes Reference Guide.

[34] Campo E.A. (2006). The Complete Part Design Handbook: For Injection Molding of Thermoplastics.

[35] Carello M., Amirth N., Airale A.G., Monti M., Romeo A. (2017). Building Block Approach for Structural Analysis of Thermoplastic Composite Components for Automotive Applications. Appl. Compos. Mater..

[36] Fuenmayor E., O’Donnell C., Gately N., Doran P., Devine D., Lyons J.G., McConville C., Major I. (2019). Mass-customization of oral tablets via the combination of 3D printing and injection molding. Int. J. Pharm..

[37] Rajamani P., Ageyeva T., Kovács J. (2021). Personalized Mass Production by Hybridization of Additive Manufacturing and Injection Molding. Polymers.

[38] Fuenmayor E., Forde M., Healy A.V., Devine D.M., Lyons J.G., McConville C., Major I. (2018). Material Considerations for Fused-Filament Fabrication of Solid Dosage Forms. Pharmaceutics.

[39] Mohamed O.A., Masood S., Bhowmik J.L. (2015). Optimization of fused deposition modeling process parameters: A review of current research and future prospects. Adv. Manuf..

[40] E Patterson A., Pereira T.R., Allison J.T., Messimer S.L. (2019). IZOD impact properties of full-density fused deposition modeling polymer materials with respect to raster angle and print orientation. Proc. Inst. Mech. Eng. Part C J. Mech. Eng. Sci..

[41] Sood A.K., Ohdar R., Mahapatra S. (2010). Parametric appraisal of mechanical property of fused deposition modelling processed parts. Mater. Des..

[42] Fuenmayor E., Forde M., Healy A.V., Devine D.M., Lyons J.G., McConville C., Major I. (2018). Comparison of fused-filament fabrication to direct compression and injection molding in the manufacture of oral tablets. Int. J. Pharm..

[43] Cao Z., Daly M., Geever L.M., Major I., Higginbotham C.L., Devine D.M. (2016). Synthesis and characterization of high density polyethylene/peat ash composites. Compos. Part B: Eng..

[44] Douglas C. (2009). Montgomery, Design and Analysis of Experiments.

[45] Cao Z., Daly M., Clémence L., Geever L.M., Major I., Higginbotham C.L., Devine D.M. (2016). Chemical surface modification of calcium carbonate particles with stearic acid using different treating methods. Appl. Surf. Sci..

[46] Ahn S.-H., Montero M., Odell D., Roundy S., Wright P.K. (2002). Anisotropic material properties of fused deposition modeling ABS. Rapid Prototyp. J..

[47] Alafaghani A., Qattawi A., Alrawi B., Guzman A. (2017). Experimental Optimization of Fused Deposition Modelling Processing Parameters: A Design-for-Manufacturing Approach. Procedia Manuf..

[48] Rodríguez-Panes A., Claver J., Camacho A.M. (2018). The Influence of Manufacturing Parameters on the Mechanical Behaviour of PLA and ABS Pieces Manufactured by FDM: A Comparative Analysis. Materials.

[49] Hossain M.S., Ramos J., Espalin D., Perez M., Wicker R. (2013). Improving Tensile Mechanical Properties of FDM-Manufactured Specimens via Modifying Build Parameters. 2013 International Solid Freeform Fabrication Symposium.

[50] Roberson D.A., Perez A.R.T., Shemelya C.M., Rivera A., MacDonald E., Wicker R.B. (2015). Comparison of stress concentrator fabrication for 3D printed polymeric izod impact test specimens. Addit. Manuf..

[51] Ma J., Zhang Y., Li J., Cui D., Wang Z., Wang J. (2021). Microstructure and mechanical properties of forging-additive hybrid manufactured Ti–6Al–4V alloys. Mater. Sci. Eng. A.

[52] Zhai W., Wang P., Ng F.L., Zhou W., Nai S.M.L., Wei J. (2021). Hybrid manufacturing of γ-TiAl and Ti–6Al–4V bimetal component with enhanced strength using electron beam melting. Compos. Part B Eng..

